# Validating the role of the Australian National University Alzheimer’s Disease Risk Index (ANU-ADRI) and a genetic risk score in progression to cognitive impairment in a population-based cohort of older adults followed for 12 years

**DOI:** 10.1186/s13195-017-0240-3

**Published:** 2017-03-04

**Authors:** Shea J. Andrews, Ranmalee Eramudugolla, Jorge I. Velez, Nicolas Cherbuin, Simon Easteal, Kaarin J. Anstey

**Affiliations:** 1 John Curtin School of Medical Research, Australian National University, Canberra, Australia; 2 Centre for Research on Ageing, Health and Wellbeing, Research School of Population Health Australian National University, The Australian National University Florey, Building 54, Mills Road, Acton ACT 2601, Canberra, Australia; 3 Universidad del Norte, Barranquilla, Colombia; 4 Neuroscience Research Group, University of Antioquia, Medellin, Colombia

**Keywords:** Alzheimer’s disease, Cognitive aging, Mild cognitive impairment (MCI), Cohort studies, Risk factors in epidemiology, Multi-state models

## Abstract

**Background:**

The number of people living with dementia is expected to exceed 130 million by 2050, which will have serious personal, social and economic implications. Employing successful intervention and treatment strategies focused on disease prevention is currently the only available approach that can have an impact on the projected rates of dementia, with risk assessment being a key component of population-based risk reduction for identification of at-risk individuals. We evaluated a risk index comprising lifestyle, medical and demographic factors (the Australian National University Alzheimer’s Disease Risk Index [ANU-ADRI]), as well as a genetic risk score (GRS), for assessment of the risk of progression to mild cognitive impairment (MCI).

**Methods:**

The ANU-ADRI was computed for the baseline assessment of 2078 participants in the Personality and Total Health (PATH) Through Life project. GRSs were constructed on the basis of 25 single-nucleotide polymorphisms previously associated with Alzheimer’s disease (AD). Participants were assessed for clinically diagnosed MCI and dementia as well as psychometric test-based MCI (MCI-TB) at 12 years of follow-up. Multi-state models were used to estimate the odds of transitioning from cognitively normal (CN) to MCI, dementia and MCI-TB over 12 years according to baseline ANU-ADRI and GRS.

**Results:**

A higher ANU-ADRI score was associated with increased risk of progressing from CN to both MCI and MCI-TB (HR 1.07 [95% CI 1.04–1.11]; 1.07 [1.04–1.09]). The GRS was associated with transitions from CN to dementia (HR 4.19 [95% CI 1.72–10.20), but not to MCI or MCI-TB (HR 1.05 [95% CI 0.86–1.29]; 1.03 [0.87–1.21]). Limitations of our study include that the ethnicity of participants in the PATH project is predominately Caucasian, potentially limiting the generalisability of the results of this study to people of other ethnicities. Biomarkers of AD were not available to define MCI attributable to AD. Not all the predictive variables for the ANU-ADRI were available in the PATH project.

**Conclusions:**

In the general population, the ANU-ADRI, comprising lifestyle, medical and demographic factors, is associated with the risk of progression from CN to MCI, whereas a GRS comprising the main AD risk genes was not associated with this risk. The ANU-ADRI may be used for population-level risk assessment and screening.

**Electronic supplementary material:**

The online version of this article (doi:10.1186/s13195-017-0240-3) contains supplementary material, which is available to authorized users.

## Background

Accurate risk assessment for cognitive impairment and dementia is increasingly important, given the current lack of effective disease-modifying treatments for Alzheimer’s disease (AD) and other dementias. Risk assessment tools may be used in both pharmacological and non-pharmacological trials, in clinics, and for population-level screening to guide risk reduction strategies [1, 2]. Validated risk assessment tools that can be administered at very low cost provide methods for clinicians in low-income countries and regions to assess dementia risk and apply prevention strategies. Given current projections of increasing dementia prevalence, there is an urgent need for validated risk assessment tools that have been evaluated in well-characterised samples over long time periods [3]. However, to our knowledge, established dementia risk tools [4] have not been evaluated for assessment of risk of mild cognitive impairment (MCI), which is a key target group for secondary prevention and pharmaceutical trials. Using a recently developed risk tool for MCI formulated in the Mayo Clinic Study of Aging, researchers found that a basic risk score composed of general demographic (e.g., age, education, marital status) and clinical (e.g., diabetes, hypertension, body mass index [BMI]) features had a c-statistic of 0.60. An augmented version containing additional variables typically collected in clinical and neurological examinations (e.g., gain speed, anxiety, Clinical Dementia Rating Sum of Boxes) had a c-statistic of 0.70 [5]. Further evaluation of this model in an independent cohort is required.

Recently, there has also been increasing interest in the evaluation of genetic risk scores (GRSs) for AD and dementia, which have been associated with the development of AD and incident MCI [6–9], though they have limited utility in predicting AD beyond that attained with basic demographic variables such as age, sex and education [7, 10, 11]. The number of studies assessing the association of AD GRS with progression between cognitive states is limited and the findings mixed. These studies include reports of a significant association between GRS and progression from cognitively normal (CN) to either MCI or late-onset Alzheimer’s disease (LOAD) with a c-statistic of 0.684 (HR 1.29 [95% CI 1.19–1.39]) [10]. Regarding the conversion from MCI to LOAD, one study found that participants harbouring 6 or more AD risk alleles progressed to AD twofold (HR 1.89 [95% CI 1.01–3.56]) more rapidly than those with only 6 alleles [12], whereas researchers in a second study observed that an AD GRS composed of 19 loci was associated with the conversion to dementia (HR 1.59 [95% CI 1.23–2.05]), but only when apolipoprotein E (*APOE*) was included in the risk score [9]. Conversely, researchers in a third study found no association between progression to dementia from MCI using an AD GRS composed of 18 loci [13].

Our study had two aims. First, we sought to evaluate the association of a non-genetic risk index with the progression from CN to cognitive impairment. Our measure [14] is a self-report risk index (the Australian National University Alzheimer’s Disease Risk Index [ANU-ADRI]) that has been externally validated in three cohorts of older adults in which it was found to be predictive of AD and dementia [15]. The second aim of the present study was to compare the ANU-ADRI with a GRS. We examined the association between cognitive impairment and the ANU-ADRI and a LOAD GRS, as assessed using a clinical criterion for MCI or dementia and psychometric test-based criteria for MCI (MCI-TB) in a community-based cohort of older adults. We first used a Cox proportional hazards model to investigate the association between the ANU-ADRI and a LOAD GRS and incident MCI/dementia, and then we extended this model using multi-state models (MSMs) to account for backward transitions between cognitive states (i.e., cognitive recovery) and competing risks (i.e., dementia and death).

## Methods

### Participants

Participants were community-dwelling adults residing in the City of Canberra, Australia, or in the neighbouring town of Queanbeyan who had been recruited into the Personality and Total Health (PATH) Through Life project, a longitudinal, population-based study of health and well-being in adults. Cohorts aged 20–24 (20+), 40–44 (40+) and 60–64 (60+) years at baseline were assessed at 4-year intervals for a total of 12 years. The background and procedures for the PATH study are described elsewhere [16]. Written informed consent was obtained from all participants. This study was approved by the human research ethics committee of The Australian National University.

In this study, we used data from the 60+ cohort with interviews conducted in 2001–2002 (*n* = 2551), 2005–2006 (*n* = 2222), 2009–2010 (*n* = 1973) and 2014–2015 (*n* = 1645). Individuals were excluded if their ethnicity was not Caucasian (*n* = 107) or if they had a self-reported history of stroke, transient ischemic attack, epilepsy, brain tumours or brain infection (*n* = 381).

### ANU-ADRI risk assessment based on demographic, lifestyle and medical risk factors

The development of the ANU-ADRI and the methodology underlying its computation have been described previously [15]. The ANU-ADRI can be computed on the basis of up to 15 predictive variables, 11 of which are available in PATH, including age (self-report), sex (self-report), alcohol consumption (calculated according to National Health and Medical Research Council 2001 guidelines [17] using number of drinks per week, with light to moderate intake in males being 0.25–20.5 drinks per week and in females being 0.25–13.5 drinks per week), education (self-reported number of years of education), diabetes (self-reported history of diabetes), depression (assessed using the Patient Health Questionnaire [PHQ-9] [18] following the coding algorithm provided in the PHQ-9 instruction manual, with a score >10 used as a cut-off), traumatic brain injury (self-reported history of traumatic brain injury with loss of consciousness), smoking (self-reported smoking status as current smoker, past smoker or never smoker), social engagement (constructed from four domains for marital status, size of social network, quality of social network, level of social activities; a fifth domain for living arrangements was not available in PATH and thus was computed pro rata as the average of the above-mentioned social engagement variables), physical activity (combined self-reported number of hours performing mild, moderate and vigorous activities, weighted by multiples of 1, 2 and 3, respectively [19]), cognitively stimulating activities (assessed as the number of cognitive activities undertaken in the last 6 months, comprising reading, writing, playing games or attending cultural events), and BMI (weight divided by height squared, expressed in kilograms per square meter). No data were available for the remaining three predictive variables: cholesterol, fish intake and pesticide exposure. The ANU-ADRI is still predictive of the development of dementia, even when a subset of variables is used [15]. Values for predictive variables included in the ANU-ADRI for PATH were selected from baseline measurements or the first occasion on which the variables were measured. To facilitate interpretation, a constant of +13 was added to the ANU-ADRI to change the range to from −13 to +19 to 0–32.

### Genotyping and genetic risk score

The most significant LOAD risk single-nucleotide polymorphisms (SNPs) identified via genome-wide association studies (GWASs) [11, 20–24] from 23 loci (*ABCA7*, *BIN1*, *CD2AP*, *CD33*, *CLU*, *CR1*, *EPHA1*, *MS4A4A*, *MS4A4E*, *MS4A6A*, *PICALM*, *HLA-DRB5*, *PTK2B*, *SORL1*, *SLC24A4-RIN3*, *DSG2*, *INPP5D*, *MEF2C*, *NME8*, *ZCWPW1*, *CELF1*, *FERMT2* and *CASS4*) were genotyped using TaqMan OpenArray assays (Life Technologies, Carlsbad, CA, USA) as previously described [25, 26], in addition to the two SNPs defining the *APOE* alleles, which were genotyped using TaqMan assays as previously described [27]. Using these LOAD risk SNPs, an explained variance-weighted genetic risk score (EV-GRS) [28] was constructed, which is the sum of all the risk alleles across the individual, weighted by minor allele frequency (MAF) and the OR associated with LOAD. The EV-GRS is calculated according to the following formula:

$$ \mathrm{E}\mathrm{V}\_\mathrm{G}\mathrm{R}\mathrm{S}=\sum_{i=1}^I\left( \log \left( O{R}_{i j}\right)\sqrt{2 M A{F}_{i j}\left(1- MA{F}_{i j}\right)}\right)\ast {G}_{i j} $$

for the *i*th patient, where $log\left({OR}_i\right)$ = the OR for the *j*th SNP, ${MAF}_{ij}$ = the MAF for the *j*th SNP, and ${G}_{ij}$ = the number of risk alleles for *j*th SNP. Individuals with missing genetic data were excluded (*n* = 240). We weighted the LOAD SNPs using the previously reported OR for LOAD and by the MAF for the CEU reference population (Utah residents with Northern and Western European ancestry; see Additional file 1: Table S1). The EV-GRS was transformed into a z-score.

### Screening and clinical assessment

The screening and clinical assessment methods at waves 1–3 are described elsewhere [29, 30] and are briefly summarised here. At each wave, the same predetermined cut-off derived from a battery of cognitive tests was used for inclusion of participants in a sub-study on mild cognitive disorders and dementia. Participants from the full cohort were selected for clinical assessment if they had any of the following: (1) a Mini Mental State Examination (MMSE) [31] score <25; (2) a score below the fifth percentile score on immediate or delayed recall of the first list of the California Verbal Learning Test [32]; or (3) a score below the fifth percentile on two or more of the Symbol Digit Modalities Test (SDMT) [33], Purdue Pegboard with both hands [34] or Simple Reaction Time [35]. At wave 4, participants were selected for review if they met any of the following criteria: (1) MMSE score <25 or ≤2.5^th^ percentile on one or more cognitive test, (2) previous diagnosis at waves 1–3, (3) subjective decline ≥25 on the Memory and Cognition Questionnaire (MACQ) or (4) decline in MMSE score ≥3 points.

The criteria for the clinical assessment for cognitive impairment at waves 1–3 has been published by our group elsewhere [30]. They involved a structured clinical assessment for dementia conducted by one of two physicians, a neuropsychological assessment, and the Clinical Dementia Rating [36], which were used together to formulate a consensus diagnosis.

**Fig. 1:**
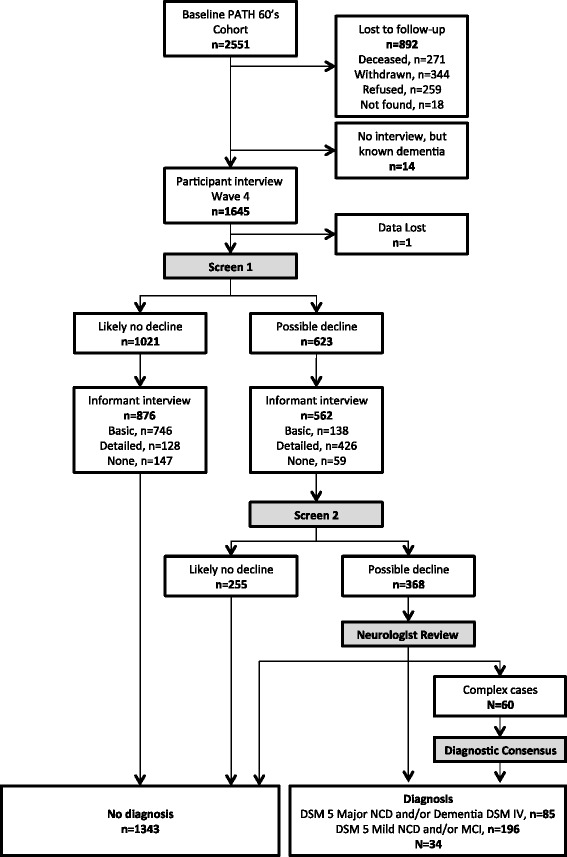
Flowchart depicting the process of screening participants for mild cognitive disorders. *DSM-IV Diagnostic and Statistical Manual of Mental Disorders, Fourth Edition*; *DSM-5 Diagnostic and Statistical Manual of Mental Disorders, Fifth Edition*; *MCI* Mild cognitive impairment; *NCD* Neurocognitive disorder; *PATH* Personality and Total Health Through Life project

Owing to the large number of participants screened for review at wave 4, diagnosis was based on neurologist review of interview data as outlined below and in Fig. 1. For each of the 1644 participants with interview data at wave 4, assessment data were screened for signs of decline on the basis of the following criteria (screen 1): a previous diagnosis of a cognitive disorder at waves 1, 2 or 3 *or* either evidence of cognitive impairment on the MMSE (≤24) or performance on one or more cognitive tests ≤6.7th percentile at wave 4 (immediate recall task, delayed recall task, SDMT, F words, A words, Boston Naming Test, Simple Response Time task, Choice Response Time task, Purdue Pegboard dominant, Purdue Pegboard non-dominant, Purdue Pegboard both, Digit Span Backward, Trail Making Test B, Stroop words, Stroop colour-word test). Additionally, participants had to show evidence of either subjective decline (score ≥25 on the MACQ [32]) or evidence of decline (>3-point decline in MMSE score since wave 3) or evidence of consistent cognitive impairment over time (MMSE ≤24 at waves 3 and 4).

All data derived from the health survey and cognitive testing as well as informant interview were collated into a spreadsheet case file for each participant. This case file (screen 2) automatically screened each participant for meeting criteria for any one of the following diagnoses: *Diagnostic and Statistical Manual of Mental Disorders, Fifth Edition* (DSM-5), major neurocognitive disorder (NCD); DSM-IV dementia; DSM-5 mild NCD; MCI; age-associated cognitive decline; age-associated memory impairment; DSM-IV amnestic disorder not otherwise specified; DSM-IV mild NCD; and DSM-IV other cognitive disorder. Major criteria for meeting most of these diagnoses were operationalised as any of the following: (1) concern of self or informant of significant cognitive decline (MACQ ≥25 *or* Informant Questionnaire on Cognitive Decline in the Elderly >3.31 *or* history of dementia diagnosis); (2) substantial impairment on at least one cognitive domain relative to wave 4 normative data (cut-offs less than −2 SD for dementias, less than −1.5 SD for mild cognitive disorders); (3) interference with independence and instrumental activities of daily living (IADL; self-reported IADL impairment *or* Bayer IADL scale score >3.11 *or* informant-reported everyday cognitive difficulties); (4) not exclusively during delirium (cognitive changes of >6 months’ duration, onset of cognitive changes preceding informant report of onset of delirium-like symptoms); and (5) not due to another co-existing disorder (PHQ9 < 9 *and* no reported history of schizophrenia or other psychosis). Those meeting criteria for one or more diagnoses (*n* = 368) were screened for case file review by a research neurologist. Diagnoses were made for 301 of these cases, of which 60 complex cases were selected for diagnostic consensus based on the following criteria: (1) comorbid depression, (2) other comorbid psychiatric conditions, (3) stroke and (4) DSM-5 major NCD without memory impairment. Following consensus diagnosis with a clinician specialising in psychiatry, the final diagnoses included 85 dementia/major NCD, 196 mild cognitive disorders (MCI/mild NCD), and 34 other mild or medically related cognitive disorders.

Clinically diagnosed MCI was based on the Petersen criteria at waves 1 and 2 [37], whereas the Winblad criteria [38] were used at waves 3 and 4. Clinically diagnosed dementia was based on the DSM-IV criteria [39] at all waves. At wave 4, there were 14 participants who were not interviewed but were known to have dementia on the basis of informant reports and medical records. Owing to the small number of individuals classified with dementia, participants with either MCI or dementia were grouped into a single MCI/dementia category.

### MCI-TB

To complement the clinical diagnosis of MCI, a broader MCI-TB classification was applied to the entire PATH sample [40] at each wave on the basis of education-adjusted cognitive performance (Table 1). The PATH sample was first stratified by education (0–12 or 13+ years). Within each of these strata, individuals were classified as MCI-TB if they scored 1.5 SD below the mean on one or more of the psychometric tests used to assess the following cognitive domains: perceptual speed measured using the SDMT [33], episodic memory assessed using the immediate recall of the first trial of the California Verbal Learning Test (recall-immediate) [32], working memory measured using the Digit Span Backward from the Wechsler Memory Scale [41] and vocabulary assessed by the Spot-the-Word test [42].

**Table 1:** Characteristics of Personality and Total Health Through Life project cohort for waves 1–4

	Wave 1, estimate ± SD	Wave 2, estimate ± SD	Wave 3, estimate ± SD	Wave 4, estimate ± SD
*n*	2078	1798	1596	1337
Age, years	63 ± 1.5	67 ± 1.5	71 ± 1.5	75 ± 1.5
Female sex, *n* (%)	1009 (48.5)	870 (48.3)	775 (48.6)	651 (48.7)
Education	14 ± 2.8	–	–	–
Wave 1 completers	12.7 ± 3.0	–	–	–
Wave 2 completers	13.1 ± 2.7	–	–	–
Wave 3 completers	13.5 ± 2.7	–	–	–
Wave 4 completers	14.2 ± 2.6	–	–	–
Immediate recall	7.2 ± 2.3	7 ± 2.2	6.7 ± 2.2	5.4 ± 1.9
Digit Span Backward	4.9 ± 2.2	5.1 ± 2.2	5.1 ± 2.2	5.3 ± 2.2
Spot-the-Word test	52.0 ± 6.0	53 ± 5.3	53 ± 5.1	54 ± 5
SDMT	50.0 ± 9.7	50 ± 9.2	48 ± 9.2	46 ± 9.5
ANU-ADRI	9.4 ± 5.9	–	–	–
EV-GRS	1.6 ± 0.4	–	–	–
Cognitive status, *n* (%)				
MCI	23 (1.1)	28 (1.6)	35 (2.2)	103 (7.7)
Dementia	0 (0)	0 (0)	7 (0.44)	37 (2.7)
MCI-TB	384 (18.4)	373 (20.7)	347 (21.7)	261 (19.5)
Attrition, *n* (%)				
Death	–	57 (2.7)	54 (2.5)	94 (5.8)
Dropout	–	280 (13.5)	167 (9.3)	329 (20.6)

*Abbreviations: ANU-ADRI* Australian National University Alzheimer’s Disease Risk Index, *EV-GRS* Explained variance-weighted genetic risk score, *SDMT* Symbol Digit Modalities Test, *MCI* Mild cognitive impairment, *MCI-TB* Test-based mild cognitive impairment

### Data analysis

All statistical analyses were performed using R version 3.1.2 software [43]. Because missing values can reduce power and introduce bias in the resulting estimates, missing values that were not attributable to attrition for the predictive variables used in the construction of the ANU-ADRI and the MCI-TB (see above) were imputed using an implementation of the random forests algorithm available in the ‘missForest’ package in R [44, 45]. This left 2078 individuals available for analysis. Additional file 2: Table S2 shows the proportion of missing variables for each variable.

We first evaluated the risk of progression from CN to MCI/dementia using Cox proportional hazards models with age as the time scale and the ANU-ADRI and EV-GRS included as predictor variables in the same model. The outcome of interest in these models was the time to first diagnosis of MCI/dementia, with those subjects who did not develop MCI/dementia at their last assessment right-censored. HRs and 95% CIs were given for the time to MCI/dementia analysis. Concordance index (c-index) for the prediction of conversion from NC to MCI/dementia was calculated. Cox proportional hazards models were estimated using the ‘survival’ package in R.

**Fig. 2:**
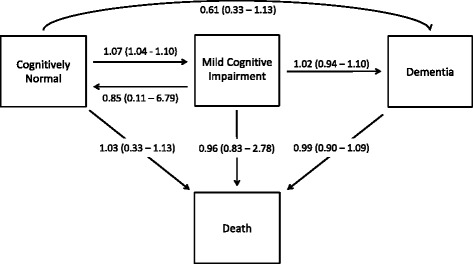
A four-state model for possible transitions between cognitive states and death. HRs (95% CIs) for the effect of the Australian National University Alzheimer’s Disease Risk Index on transitions between cognitively normal, mild cognitive impairment, dementia and death are shown. All estimates are from models adjusting for the explained variance-weighted genetic risk score

To evaluate a more complex model of disease progression, MSMs were used to examine the association between the ANU-ADRI and EV-GRS and transitions between cognitive states. MSMs allow the modelling of competing risks and backward transitions between states (i.e., recovery) [46]. Hidden Markov models can be used to estimate misclassification error, and the effects of covariates can be allowed to vary by transition [46]. The MSMs used in this analysis modelled cognitive deterioration and cognitive recovery by allowing transitions and backward transitions between CN, MCI or MCI-TB states. Backward transitions from dementia were not allowed, whereas death was used as a fourth absorbing state (Fig. 2). Individuals with only a single observation (i.e., no recorded transitions) were excluded from the analysis (*n* = 204). Individuals lost to attrition were considered right-censored. The ANU-ADRI and the EV-GRS were included as covariates in the same model. Maximum likelihood estimates of parameters in the MSMs were obtained with the Broyden-Fletcher-Goldfarb-Shanno optimisation method. Normalisation was applied to the likelihood function to improve numerical stability. Because the likelihood is maximised using numerical methods, an input of initial values is required to start the search for a maximum. MSMs were fitted using ‘msm’ [46] in R, and multiple models were run using different sets of initial values to ensure the robustness of the parametric estimates. See Additional file 3 for more details on the structure of MSMs.

As a sensitivity analysis for the MCI-TB analysis, more stringent criteria were investigated with MCI-TB based on a score of 1.5 SD below the mean on two or more of the above-mentioned psychometric tests. Additionally, we performed a complete case analysis to ensure that our imputation method was not biasing the observed results.

## Results

### Demographics and other characteristics of the sample

**Fig. 3:**
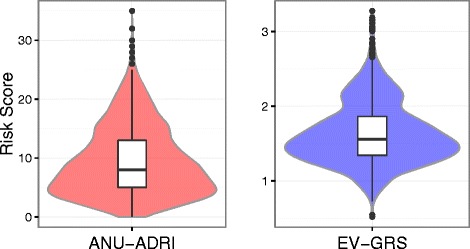
Distribution of the Australian National University Alzheimer’s Disease Risk Index (ANU-ADRI) and explained variance-weighted genetic risk score (EV-GRS) within the Personality and Total Health Through Life project cohort. The variable width of the violin plot indicates the probability density, and the box plot indicates the first, median and third quartiles of the ANU-ADRI and EV-GRS scores

Baseline distributions of education, depression, sex, ANU-ADRI, raw cognitive tests scores and cognitive states at each wave for the PATH cohort are described in Table 1. Participants who completed all four waves of interviews had a higher level of education than participants who completed only the wave 1 interview (*t* = −6.8, *df* = 331.3, *p* < 0.001). Participants were followed for an average of 9.6 years (after accounting for loss due to attrition) and a total of 13.9 years. Group differences in the sub-indices of the ANU-ADRI between CN and either MCI/dementia or MCI-TB can be found in Additional file 4: Table S3. The distribution of the ANU-ADRI and EV-GRS scores is shown in Fig. 3. As expected, the proportion of individuals classified as MCI/dementia increased over the course of the study, whereas the proportion of individuals classified as MCI-TB remained stable (Table 1). By wave 4, 36% of the cohort had been lost to follow-up, with 57, 54 and 94 individuals deceased by waves 2, 3 and 4, respectively, and an additional 280, 267 and 329 individuals being lost to follow-up for other reasons (e.g., refusal, left catchment area) at waves 2, 3 and 4, respectively.

Between any two waves, a greater proportion of people transitioned from CN to MCI-TB (10.5%) than from unimpaired to MCI (2.6%), indicating that MCI-TB is a broader categorisation of cognitive impairment. A smaller proportion of individuals transitioned in the opposite direction—from MCI-TB to CN (31.3%)—than from MCI to CN (44%), indicating that MCI-TB is also a more stable category (Table 2).

**Table 2:** Number of transitions between cognitively normal, mild cognitive impairment, dementia and test-based mild cognitive impairment during the length of the study

To					
From	CN	MCI	Dementia	Death	Censored
MCI and dementia					
CN	4459 (86.1%)	137 (2.6%)	32 (0.6%)	189 (3.7%)	359 (6.9%)
MCI	40 (48.2%)	26 (31.3%)	6 (7.2%)	5 (6%)	6 (7.2%)
Dementia	0 (0%)	0 (0%)	5 (71.4%)	2 (28.6%)	0 (0%)
Censored	36 (19.5%)	3 (1.6%)	0 (0%)	8 (4.3%)	138 (74.6%)
MCI-TB					
CN	3403 (80.2%)	446 (10.5%)		144 (3.4%)	249 (5.9%)
MCI	321 (31.3%)	524 (51.2%)		52 (5.1%)	127 (12.4%)
Censored	28 (15.1%)	11 (5.9%)		8 (4.3%)	138 (74.6%)

*Abbreviations: CN* Cognitively normal, *MCI* Mild cognitive impairment, *MCI-TB* Test-based mild cognitive impairment

### Cox proportional hazards models for incident MCI

A higher ANU-ADRI (indicating greater risk) score was associated with an increased risk of progression to both MCI/Dementia and MCI-TB (Table 3). The EV-GRS was not associated with progression to either MCI/Dementia or MCI-TB. The interaction between the ANU-ADRI and the EV-GRS was non-significant for the MCI/Dementia (HR 0.99 [95% CI 0.96–1.01], *p* = 0.33) and MCI-TB (HR 0.99 [95% CI 0.98–1.01], *p* = 0.11).

**Table 3:** Associations between the Australian National University Alzheimer’s Disease Risk Index and explained variance-weighted genetic risk scores and cognitive impairment at waves 1–4

	MCI/dementia	MCI-TB
ANU-ADRI^a^, HR (95% CI)	1.06 (1.03–1.09)^b^	1.04 (1.02–1.50)^b^
EV-GRS^c^, HR (95% CI)	1.14 (0.98–1.33)	1.04 (0.96–1.12)
C-index (SE)	0.61 (0.03)	0.56 (0.01)
ANU-ADRI	0.60 (0.05)	0.56 (0.02)
EV-GRS	0.53 (0.05)	0.51 (0.02)

*Abbreviations: ANU-ADRI* Australian National University Alzheimer’s Disease Risk Index; *C-index* Concordance index; *EV-GRS* Explained variance-weighted genetic risk score

All estimates are derived from models adjusting for ANU-ADRI and EV-GRS

^a^Per unitary increase in ANU-ADRI

^b^ *p* < .001

^c^Per SD increase in EV-GRS

In the sensitivity analysis, using a more stringent MCI-TB criterion (scoring 1.5 SD below the mean on two or more tests) confirmed that the ANU-ADRI was associated an increased risk of progression from CN to MCI-TB (HR 1.08 [95% CI 1.05–1.10], *p* = < 0.0001). In the complete case analysis, the ANU-ADRI remained significant for both the MCI/dementia (HR 1.06 [95% CI 1.02–1.09], *p =* 0.001) and MCI-TB (HR 1.036 [95% CI 1.01–1.04], *p =* 0.007) models.

### Multi-state models of transitions

A higher ANU-ADRI score was associated with an increased risk of transitioning from CN to MCI or MCI-TB (Fig. 1, Table 4). The probability of transitioning from CN to either MCI or MCI-TB after 12 years for individuals scoring 1 SD below the mean on the ANU-ADRI was 10%, and for individuals scoring 1 SD above the mean, the probability of transitioning was 20%. A higher ANU-ADRI score was not associated with transitions from CN, MCI, dementia or MCI-TB to death; with transition from cognitive impairment to dementia; or with cognitive recovery from MCI or MCI-TB to CN. The EV-GRS was associated with an increased risk of transitioning from CN to dementia, with the probability of transitioning from CN to dementia for individuals scoring 1 SD above the mean being 1.3%. The interaction between the ANU-ADRI and the EV-GRS was not significant for any of the transitions for either the MCI or MCI-TB models.

**Table 4:** HRs (95% CIs) of the Australian National University Alzheimer’s Disease Risk Index and explained variance-weighted genetic risk scores upon cognitive transition

Transition	MCI and dementia		MCI-TB	
	ANU-ADRI^a^	EV-GRS^b^	ANU-ADRI^a^	EV-GRS^b^
CN to MCI	1.07 (1.04–1.10)^c^	1.05 (0.86–1.29)	1.07 (1.04–1.09)^c^	1.03 (0.87–1.21)
CN to dementia	0.61 (0.33–1.13)	4.19 (1.72–10.2)^c^		
CN to death	1.03 (0.94–1.12)	0.70 (0.27–1.84)	1.02 (0.98–1.06)	0.89 (0.69–1.16)
MCI to CN	0.85 (0.11–6.79)	0.95 (0–181.21)	0.71 (0.50–1.00)	0.44 (0.12–1.54)
MCI to dementia	1.02 (0.94–1.10)	1.19 (0.76–1.85)		
MCI to death	0.96 (0.83–1.11)	0.87 (0.29–2.63)	1.05 (0.98–1.12)	1.05 (0.65–1.71)
Dementia to death	0.99 (0.90–1.09)	0.78 (0.51–1.19)		

*Abbreviations: ANU-ADRI* Australian National University Alzheimer’s Disease Risk Index, *EV-GRS* Explained variance-weighted genetic risk score, *CN* Cognitively normal, *MCI/dementia* Mild cognitive impairment or dementia, *MCI-TB* Test-based mild cognitive impairment

All estimates are derived from models adjusting for the ANU-ADRI and EV-GRS

^a^Per unitary increase in ANU-ADRI

^b^Per SD increase in EV-GRS

^c^ *p* < 0.05

In the sensitivity analysis, using a more stringent MCI-TB criterion (Additional file 5: Table S4) confirmed that the ANU-ADRI was associated an increased risk of progression from CN to MCI-TB (HR 1.12 [95% CI 1.07–1.17]). For the complete case analysis (Additional file 6: Table S5), the ANU-ADRI remained statistically significant for both the models for transition from CN to MCI (1.06 [1.02–1.09]) and from CN to MCI-TB (HR 1.05 [95% CI 1.01–1.08]).

## Discussion

To our knowledge, we report the first concurrent evaluation of a non-genetic risk score and a GRS in the risk of progression to MCI over a long period in a population-based cohort. As such, this study provides much-needed information on the utility of risk assessment tools in evaluating the risk of progression to MCI in the general population. Using Cox proportional hazards models, we found that a unitary increase in the ANU-ADRI at baseline was associated with 6% and 4% increased hazards of transitioning from CN to MCI/dementia and MCI-TB, respectively. Additionally, we used MSMs to extend the Cox proportional hazards models to account for backward transitions between cognitive states and the competing risks of death and dementia. We observed that a unitary change in the ANU-ADRI was associated with a 7% increased hazard of transitioning from CN to either MCI or MCI-TB. In contrast, the EV-GRS was not associated with transition from CN to cognitive impairment, though it was associated with a 419% increased hazard of transitioning to dementia from CN.

MSMs are well suited to analysing a more ‘realistic’ model of cognitive decline in which cognitive deterioration and recovery are modelled simultaneously in addition to misclassification, death and censoring. This is important in the examination of MCI because pathological cognitive change is often not a linear progression from CN to MCI and finally to dementia; reversions from MCI back to CN are common, which was also observed in the PATH cohort [30, 47]. Individuals with a stable progression to MCI are more likely to progress to dementia than those with an unstable course or no diagnosis of MCI [47]. A higher ANU-ADRI score is associated both with an increased risk of transition to clinically diagnosed MCI and to MCI-TB, suggesting that it could be useful for assessing an individual’s risk of developing MCI. Additionally, even in individuals who revert to CN, the diagnosis of cognitive impairment may still have prognostic implications because these individuals have a greater likelihood of progressing to dementia or MCI than those who remain CN [47]. As such, individuals with a higher ANU-ADRI are more likely to revert to MCI or develop dementia in the future [15]. These results show that the ANU-ADRI may be used to measure risk reduction for clinically significant MCI as well as dementia, and it may have implications for secondary prevention of dementia. However, although the ANU-ADRI is strongly associated with the progression from CN to MCI, its predictive ability was limited (c-index 0.60 for MCI and 0.56 for MCI-TB). This may be due to the relatively young age of the PATH cohort and consequently the small number of participants with MCI and the narrow age range of the sample. We expect that further validation of the ANU-ADRI in a slightly older cohort with a higher incidence of MCI or with a wider age range would show that the ANU-ADRI has greater predictive ability.

The ANU-ADRI has several strengths [4]. First, the ANU-ADRI is the only risk assessment tool that has not been developed by identifying risk factors through the analysis of a single cohort, and as such the predictive variables are not optimised to a particular study. The ANU-ADRI also does not include any risk factors that require clinical assessments or laboratory tests.

The genetic risk score was observed to be associated with the transition from CN to dementia, but not with the transition from CN to MCI or from MCI to dementia. This lack of an association may be a result of the broad categorisation of MCI rather than MCI subtypes, such that it would have included participants with cognitive impairment that was not MCI due to AD [48, 49]. This may also explain the reduced risk associated with both MCI and MCI-TB in our sensitivity analysis. Unfortunately, owing to the small number of participants with MCI in the PATH cohort, further subgroup analysis would likely be underpowered to detect an effect. However, it should be noted that most dementia cases are associated with mixed pathologies rather than singular pathologies, suggesting that an AD GRS would be associated with both amnestic and non-amnestic MCI [50].

Researchers in previous studies have investigated the association of AD GRS with MCI. In 3605 participants (360 MCI, 191 dementia), an AD GRS composed of *APOE* + 19 LOAD GWAS variants was associated with an increased risk of incident MCI and nominally associated with amnestic and non-amnestic cases [9]. In a second study of 2674 participants (347 MCI, 132 LOAD), a GRS composed of *APOE* + nine LOAD GWAS variants was associated with progression from CN to MCI/LOAD [10]. Lack of replication in this study could be due to younger and fewer cognitively impaired participants. Furthermore, inclusion of additional AD risk loci that were identified to be nominally significant in relation to AD in GWASs may identify a stronger association [8].

Limitations of our study include the relatively high level of education of the PATH cohort [16]. Also, the ethnicity distribution in the PATH cohort is predominately Caucasian, potentially limiting the generalisability of the results of this study to other ethnicities, and biomarkers of AD were not available (e.g., cerebrospinal fluid, amyloid-β). Not all the predictive variables for the ANU-ADRI were available in PATH, suggesting that the present study may underestimate the sensitivity of this tool in predicting individuals who are at risk of developing cognitive impairment. However, the validation studies also included a subset of the variables contributing to the ANU-ADRI [15].

Study strengths included the large population-based sample with high retention rates and 12 years of follow-up data. The PATH cohort was recruited from a narrow age band, reducing the impact of age differences on the findings. This is particularly important because age has the largest weighting of risk factors in the ANU-ADRI. Finally, the conservative clinical classifications of MCI/dementia, based on a thorough clinical assessment and consensus diagnosis by clinicians using published criteria, were complemented by a broader classification of MCI (MCI-TB).

## Conclusions

Higher ANU-ADRI scores are associated with increased risk of progressing from CN to MCI. These results complement previous evidence that the ANU-ADRI is predictive of AD and dementia [15]. In comparison, a GRS comprising the main AD genes was associated with the development of dementia but was not associated with the risk of developing MCI. These results provide further support for using the ANU-ADRI for population-level strategies, individual patient assessment, and for informing intervention and treatment strategies aimed at delaying or preventing dementia.

## Additional files

Additional file 1: Table S1. LOAD risk SNPs used in this study. (DOCX 89 kb)
Additional file 2: Table S2. Proportion of missing data that was imputed for ADRI sub-indices and cognitive variables. (DOCX 93 kb)
Additional file 3: Supplementary methods. Further detail on multi-state models. (DOCX 93 kb)
Additional file 4: Table S3. Differences between NC and individuals classified as MCI/dementia or MCI-TB at any wave for the ANU-ADRI, ANU-ADRI sub-indices and the EV-GRS. (DOCX 91 kb)
Additional file 5: Table S4. Hazard ratios (95% CIs) of the ANU-ADRI and EV-GRS scores upon cognitive transition in the MCI-TB sensitivity analysis using a more stringent criterion of MCI-TB (scoring 1.5 SD below the mean on two or more tests). (DOCX 51 kb)
Additional file 6: Table S5. Hazard ratios (95% CIs) of the ANU-ADRI and EV-GRS scores upon cognitive transition in the complete case sensitivity analysis. (DOCX 65 kb)

## Abbreviations

AD: Alzheimer’s disease
ANU-ADRI: Australian National University Alzheimer’s Disease Risk Index
*APOE*: *Apolipoprotein E*
BMI: Body mass index
c-index: Concordance index
CN: Cognitively normal
DSM-5: *Diagnostic and Statistical Manual of Mental Disorders, Fifth Edition*
DSM-IV: *Diagnostic and Statistical Manual of Mental Disorders, Fourth Edition*
EV-GRS: Explained variance-weighted genetic risk score
GRS: Genetic risk score
GWAS: Genome-wide association study
IADL: Instrumental activities of daily living
LOAD: Late-onset Alzheimer’s disease
MACQ: Memory and Cognition Questionnaire
MAF: Minor allele frequency
MCI: Mild cognitive impairment
MCI-TB: Test-based mild cognitive impairment
MMSE: Mini Mental State Examination
MSM: Multi-state model
NCD: Neurocognitive disorder
NHMRC: National Health and Medical Research Council
PATH: Personality and Total Health Through Life project
PHQ-9: Patient Health Questionnaire
SDMT: Symbol Digit Modalities Test
SNP: Single-nucleotide polymorphism
