# Supplementary material for: Validating the role of the Australian National University Alzheimer’s Disease Risk Index (ANU-ADRI) and a genetic risk score in progression to cognitive impairment in a population-based cohort of older adults followed for 12 years
Source: Alzheimers Res Ther. 2017 Mar 4;9:16. doi: 10.1186/s13195-017-0240-3 (PMC5336661; doi:10.1186/s13195-017-0240-3)
Supplement: Additional file 1: Table S1. — LOAD risk SNPs used in this study. (DOCX 89 kb) [file 13195_2017_240_MOESM1_ESM.docx]

**Table S1:** LOAD risk SNPs used in this study

| **Gene** | **SNP** | **Chromosome** | **Alleles**^*^ | **MAF**^†^ | **OR**^‡^ |
| --- | --- | --- | --- | --- | --- |
| *APOE ε4* | rs429358/rs7412 | 19 | ε2/ε3/ε4 | 0.8/0.14 | 0.54/3.81 |
| *ABCA7* | rs3764650 | 19 | T/G | 0.11 | 1.23 |
| *BIN1* | rs744373 | 2 | A/G | 0.31 | 1.17 |
| *CD2AP* | rs9296559 | 6 | T/C | 0.27 | 1.11 |
| *CD33* | rs34813869 | 19 | A/G | 0.3 | 0.89 |
| *CLU* | rs11136000 | 8 | C/T | 0.35 | 0.88 |
| *CR1* | rs3818361 | 1 | G/A | 0.26 | 1.17 |
| *EPHA1* | rs11767557 | 7 | T/C | 0.2 | 0.89 |
| *MS4A4A* | rs4938933 | 11 | T/C | 0.5 | 0.88 |
| *MS4A4E* | rs670139 | 11 | G/T | 0.34 | 1.08 |
| *MS4A6A* | rs610932 | 11 | T/G | 0.45 | 0.90 |
| *PICALM* | rs3851179 | 11 | C/T | 0.41 | 0.88 |
| *HLA-DRB5*  *HLA-DRB1* | rs9271100 | 6 | C/T | 0.31 | 1.11 |
| *PTK2B* | rs28834970 | 8 | T/C | 0.32 | 1.10 |
| *SORL1* | rs11218343 | 11 | T/C | 0.03 | 0.77 |
| *SLC24A4-RIN3* | rs10498633 | 14 | G/T | 0.19 | 0.91 |
| *DSG2* | rs8093731 | 18 | *C/T* | 0.01 | 0.73 |
| *INPP5D* | rs35349669 | 2 | C/T | 0.44 | 1.08 |
| *MEF2C* | rs304132 | 5 | G/A | 0.46 | 0.93 |
| *NME8* | rs2718058 | 7 | A/G | 0.36 | 0.93 |
| *ZCWPW1* | rs1476679 | 7 | T/C | 0.32 | 0.91 |
| *CELF1* | rs7933019 | 11 | G/C | 0.34 | 1.08 |
| *FERMT2* | rs17125944 | 14 | T/C | 0.08 | 1.14 |
| *CASS4* | rs7274581 | 20 | T/C | 0.11 | 0.88 |

^*^Major/Minor Allele; ^†^Minor Allele Frequency: HapMap-CEU; ^‡^Alzegene [1] reported OR for minor allele or OR reported in [2]

1. Bertram L, McQueen MB, Mullin K, Blacker D, Tanzi RE. Systematic meta-analyses of Alzheimer disease genetic association studies: the AlzGene database. Nat Genet. 2007;39:17–23.

2. Lambert JC, Ibrahim-Verbaas CA, Harold D, Naj AC, Sims R, Bellenguez C, et al. Meta-analysis of 74,046 individuals identifies 11 new susceptibility loci for Alzheimer's disease. Nat Genet. 2013;45:1452–8.
