# Supplementary material for: Validating the role of the Australian National University Alzheimer’s Disease Risk Index (ANU-ADRI) and a genetic risk score in progression to cognitive impairment in a population-based cohort of older adults followed for 12 years
Source: Alzheimers Res Ther. 2017 Mar 4;9:16. doi: 10.1186/s13195-017-0240-3 (PMC5336661; doi:10.1186/s13195-017-0240-3)
Supplement: Additional file 2: Table S2. — Proportion of missing data that was imputed for ADRI sub-indices and cognitive variables. (DOCX 93 kb) [file 13195_2017_240_MOESM2_ESM.docx]

**Table S2:** Proportion of missing data that was imputed for ADRI sub-indices and cognitive variables

| Variable | Complete, n (%) | Missing, n (%) |
| --- | --- | --- |
| ADRI | 1497 (58.68) | 1054 (41.32) |
| Alcohol Intake^†^ | 2543 (99.69) | 8 (0.31) |
| Age/Sex^†^ | 2551 (100.00) | 0 (0.00) |
| Education^†^ | 2423 (94.98) | 128 (5.02) |
| Diabetes^†^ | 2547 (99.84) | 4 (0.16) |
| Depression^†^ | 2539 (99.53) | 12 (0.47) |
| Traumatic Brain Injury^†^ | 2547 (99.84) | 4 (0.16) |
| BMI | 2523 (98.90) | 28 (1.10) |
| Height^†^ | 2322 (91.02) | 229 (8.98) |
| Weight^†^ | 2547 (99.84) | 4 (0.16) |
| Smoking^†^ | 2517 (98.67) | 34 (1.33) |
| Social Engagement | 2548 (99.88) | 3 (0.12) |
| Social Support^†^ | 1964 (76.99) | 587 (23.01) |
| Marital Status^†^ | 2538 (99.49) | 13 (0.51) |
| Social Network^‡^ | 2249 (88.16) | 302 (11.84) |
| Social Activities^†^ | 2538 (99.49) | 13 (0.51) |
| Physical Activity^†^ | 2537 (99.45) | 14 (0.55) |
| Cognitive Activity | 2538 (99.49) | 13 (0.51) |
| Reading^†^ | 2538 (99.49) | 13 (0.51) |
| Writing ^†^ | 2423 (94.98) | 128 (5.02) |
| Playing Games^†^ | 2547 (99.84) | 4 (0.16) |
| Attending cultural events^†^ | 2539 (99.53) | 12 (0.47) |
| Immediate Recall |  |  |
| Wave 1 | 2551 (100) | 0 (0) |
| Wave 2 | 2182 (98.2) | 40 (1.8) |
| Wave 3 | 1949 (98.78) | 24 (1.22) |
| Wave 4 | 1625 (98.78) | 20 (1.22) |
| Digits Backwards |  |  |
| Wave 1 | 2543 (99.69) | 8 (0.31) |
| Wave 2 | 2159 (97.16) | 63 (2.84) |
| Wave 3 | 1925 (97.57) | 48 (2.43) |
| Wave 4 | 1604 (97.51) | 41 (2.49) |
| Spot-the-Word |  |  |
| Wave 1 | 2485 (97.41) | 66 (2.59) |
| Wave 2 | 2088 (93.97) | 134 (6.03) |
| Wave 3 | 1845 (93.51) | 128 (6.49) |
| Wave 4 | 1466 (89.12) | 179 (10.88) |
| Symbol Digits Modalities test |  |  |
| Wave 1 | 2531 (99.22) | 20 (0.78) |
| Wave 2 | 2178 (98.02) | 44 (1.98) |
| Wave 3 | 1909 (96.76) | 64 (3.24) |
| Wave 4 | 1511 (91.85) | 134 (8.15) |

^†^Baseline measure; ^‡^Wave 3 measure
