# Supplementary material for: Validating the role of the Australian National University Alzheimer’s Disease Risk Index (ANU-ADRI) and a genetic risk score in progression to cognitive impairment in a population-based cohort of older adults followed for 12 years
Source: Alzheimers Res Ther. 2017 Mar 4;9:16. doi: 10.1186/s13195-017-0240-3 (PMC5336661; doi:10.1186/s13195-017-0240-3)
Supplement: Additional file 3: — Supplementary methods. Further detail on multi-state models. (DOCX 93 kb) [file 13195_2017_240_MOESM3_ESM.docx]

**Supplementary methods**

The central structure of the multistate models (MSMs) is the transition intensity matrix *Q*, an *n* x *n* matrix that defines which transitions can occur.

$$Q= \left( \begin{matrix} -\left( q_{12}+q_{13} \right) & q_{12} & q_{13} \\ q_{21} & -\left( q_{21}+q_{23} \right) & q_{23} \\ 0 & 0 & 0 \end{matrix} \right)$$

Each element of the *Q*, denoted as *q_rs_*, represents the instantaneous risk of transitioning from state *r* to state *s* (i.e., the hazard ratio [HR] from *r* to *s*, with a value of 0 representing a position from which no transitions are possible [i.e., an absorbing state]). Each row of the *Q* sums to 0. For a continuous-time homogeneous Markov process with transition intensity matrix *Q*, it is possible to compute a transition probability matrix *P* over a given period of time. The likelihood of transitioning from state *r* to state *s* is conditional on the state currently occupied, but not on any state prior and assumes that transition intensities are constant over time [1]. Transition probabilities can be further modelled as a function of covariates via proportional hazard models to estimate the HR from state *r* to state *s* in the presence of a covariates. Within a multi-state model, misclassification error can be accounted for using a hidden Markov model where the observed states are assumed to be misclassifications of their true, hidden state [1]. Here the Markov process in *Q* governs the progression through the hidden states and the observed states are generated from the hidden states according to a misclassification probability matrix *E*, an analogous *n* x *n* matrix to *Q* that determines the probability of a state been erroneously classified as another state.

1. Jackson C. Multi-state modelling with R: the msm package. J Stat Soft. 2011;38:1–29.
