# Supplementary material for: Validating the role of the Australian National University Alzheimer’s Disease Risk Index (ANU-ADRI) and a genetic risk score in progression to cognitive impairment in a population-based cohort of older adults followed for 12 years
Source: Alzheimers Res Ther. 2017 Mar 4;9:16. doi: 10.1186/s13195-017-0240-3 (PMC5336661; doi:10.1186/s13195-017-0240-3)
Supplement: Additional file 4: Table S3. — Differences between NC and individuals classified as MCI/dementia or MCI-TB at any wave for the ANU-ADRI, ANU-ADRI sub-indices and the EV-GRS. (DOCX 91 kb) [file 13195_2017_240_MOESM4_ESM.docx]

**Table S3:** Differences between NC and individuals classified as MCI/Dementia or MCI-TB at any wave for the ANU-ADRI, ANU-ADRI subindices and the EV-GRS.

|  |  | | MCI/Dementia | | | MCI-TB | | |
| --- | --- | --- | --- | --- | --- | --- | --- | --- |
|  | Score | | CN | Impaired | p | CN | Impaired | p |
| EV-GRS^*^ | |  | 1.6 ± 0.41 | 1.7 ± 0.43 | 0.1188 | 1.6 ± 0.42 | 1.6 ± 0.41 | 0.5165 |
| ADRI^*^ | |  | 9.2 ± 5.8 | 11 ± 6.5 | 0.0003 | 8.8 ± 5.6 | 10 ± 6.3 | 1.068e-08 |
| **Alcohol Intake**^†^ | | | | | 0.38 |  |  | 0.22 |
| Light - Mod | -3 | | 1540 (81) | 148 (79) |  | 1074 (82) | 614 (80) |  |
| No intake | 0 | | 350 (19) | 40 (21) |  | 235 (18) | 155 (20) |  |
| **Age/Sex**^†^ | | |  |  | 0.49 |  |  | 0.45 |
| <65 years | 0 | | 1884 (100) | 187 (99) |  | 1306 (100) | 765 (99) |  |
| 65-70 males | 1 | | 2 (0.11) | 0 (0) |  | 1 (0.076) | 1 (0.13) |  |
| 65-70 females | 5 | | 4 (0.21) | 1 (0.53) |  | 2 (0.15) | 3 (0.39) |  |
| **Education**^†^ | | |  |  | 0.004 |  |  | 1.70e-05 |
| > 11 years | 0 | | 1469 (78) | 128 (68) |  | 999 (76) | 598 (78) |  |
| 8 - 11 years | 3 | | 387 (20) | 52 (28) |  | 297 (23) | 142 (18) |  |
| < 8 years | 6 | | 34 (1.8) | 8 (4.3) |  | 13 (0.99) | 29 (3.8) |  |
| **Diabetes**^†^ | | |  |  | 0.027 |  |  | 0.22 |
| No Diabetes | 0 | | 1759 (93) | 166 (88) |  | 1220 (93) | 705 (92) |  |
| Diabetes | 3 | | 131 (6.9) | 22 (12) |  | 89 (6.8) | 64 (8.3) |  |
| **Depression**^†^ | | |  |  | 0.025 |  |  | 0.00048 |
| PHQ < 10 | 0 | | 1838 (97) | 177 (94) |  | 1283 (98) | 732 (95) |  |
| PHQ > 10 | 2 | | 52 (2.8) | 11 (5.9) |  | 26 (2) | 37 (4.8) |  |
| **Traumatic Brain Injury**^†^ | | | | | 0.61 |  |  | 0.41 |
| No TBI | 0 | | 1793 (95) | 177 (94) |  | 1245 (95) | 725 (94) |  |
| TBI | 4 | | 97 (5.1) | 11 (5.9) |  | 64 (4.9) | 44 (5.7) |  |
| **Body Mass Index**^†^ |  | |  |  | 0.096 |  |  | 0.78 |
| BMI < 25 | 0 | | 685 (36) | 67 (36) |  | 467 (36) | 285 (37) |  |
| BMI 25 - 30 | 2 | | 870 (46) | 76 (40) |  | 603 (46) | 343 (45) |  |
| BMI > 30 | 5 | | 335 (18) | 45 (24) |  | 239 (18) | 141 (18) |  |
| **Smoking**^†^ | | |  |  | 0.34 |  |  | 0.84 |
| Never Smoker | 0 | | 989 (52) | 108 (57) |  | 697 (53) | 400 (52) |  |
| Past Smoker | 1 | | 700 (37) | 65 (35) |  | 476 (36) | 289 (38) |  |
| Current Smoker | 4 | | 201 (11) | 15 (8) |  | 136 (10) | 80 (10) |  |
| **Social Engagement**^†^ | | |  |  | 0.68 |  |  | 0.01 |
| Highest | 0 | | 1162 (61) | 111 (59) |  | 834 (64) | 439 (57) |  |
| Medium - Low | 1 | | 556 (29) | 57 (30) |  | 364 (28) | 249 (32) |  |
| Low | 6 | | 172 (9.1) | 20 (11) |  | 111 (8.5) | 81 (11) |  |
| **Physical Activity**^†^ |  | |  |  | 0.42 |  |  | 0.01 |
| High | -3 | | 237 (13) | 18 (9.6) |  | 167 (13) | 88 (11) |  |
| Medium | -2 | | 615 (33) | 59 (31) |  | 451 (34) | 223 (29) |  |
| Low | 0 | | 1038 (55) | 111 (59) |  | 691 (53) | 458 (60) |  |
| **Cognitive Activity**^†^ | | |  |  | 0.00072 |  |  | 4.60e-16 |
| Medium | -7 | | 743 (39) | 64 (34) |  | 529 (40) | 278 (36) |  |
| High | -6 | | 689 (36) | 54 (29) |  | 526 (40) | 217 (28) |  |
| Low | 0 | | 458 (24) | 70 (37) |  | 254 (19) | 274 (36) |  |

^*^Unpaired two tailed t-test; ^†^Fishers Exact test
