# Supplementary material for: Validating the role of the Australian National University Alzheimer’s Disease Risk Index (ANU-ADRI) and a genetic risk score in progression to cognitive impairment in a population-based cohort of older adults followed for 12 years
Source: Alzheimers Res Ther. 2017 Mar 4;9:16. doi: 10.1186/s13195-017-0240-3 (PMC5336661; doi:10.1186/s13195-017-0240-3)
Supplement: Additional file 5: Table S4. — Hazard ratios (95% CIs) of the ANU-ADRI and EV-GRS scores upon cognitive transition in the MCI-TB sensitivity analysis using a more stringent criterion of MCI-TB (scoring 1.5 SD below the mean on two or more tests). (DOCX 51 kb) [file 13195_2017_240_MOESM5_ESM.docx]

**Table S4:** Hazard ratios (95% CI) of the ANU-ADRI and EV-GRS scores upon cognitive transition in the MCI-TB sensitivity analysis using a more stringent criterion of MCI-TB (scoring 1.5 SD below the mean on two or more test)

| **Transition** | **MCI-TB** | |
| --- | --- | --- |
|  | ANU-ADRI^†^ | EV-GRS^‡^ |
| **CN - MCI** | 1.12 (1.07 - 1.17) | 0.96 (0.69 - 1.33) |
| **CN - Death** | 1.03 (1 - 1.06) | 0.98 (0.81 - 1.18) |
| **MCI - CN** | 1.22 (0.94 - 1.59) | 6.27 (0.63 - 62.59) |
| **MCI - Death** | 1 (0.93 - 1.08) | 0.78 (0.36 - 1.70) |

*p < .05;. CN: Cognitively normal; MCI/Dementia: Mild cognitive impairment or Dementia; MCI-TB: Test-based mild cognitive impairment; ^†^per unitary increase in the ANU-ADRI; ^‡^per SD increase in EV-GRS; all estimates are from models adjusting for the ANU-ADRI and EV-GRS
