# Supplementary material for: Validating the role of the Australian National University Alzheimer’s Disease Risk Index (ANU-ADRI) and a genetic risk score in progression to cognitive impairment in a population-based cohort of older adults followed for 12 years
Source: Alzheimers Res Ther. 2017 Mar 4;9:16. doi: 10.1186/s13195-017-0240-3 (PMC5336661; doi:10.1186/s13195-017-0240-3)
Supplement: Additional file 6: Table S5. — Hazard ratios (95% CIs) of the ANU-ADRI and EV-GRS scores upon cognitive transition in the complete case sensitivity analysis. (DOCX 65 kb) [file 13195_2017_240_MOESM6_ESM.docx]

**Table S5:** Hazard ratios (95% CI) of the ANU-ADRI and EV-GRS scores upon cognitive transition in the complete case sensitivity analysis

| **Transition** | **MCI and Dementia** | | **MCI-TB** | |
| --- | --- | --- | --- | --- |
|  | ANU-ADRI^†^ | EV-GRS^‡^ | ANU-ADRI^†^ | EV-GRS^‡^ |
| **CN - MCI** | **1.06 (1.02 - 1.09)*** | 1.05 (0.86 - 1.28) | **1.05 (1.01 - 1.08)*** | 1.09 (0.91 - 1.3) |
| **CN - Dementia** | 1.00 (0.02 - 62.79) | 0.84 (0 – 5.80e+11) |  |  |
| **CN - Death** | 1.00 (0.02 - 57.02) | 0.84 (0 – 3.80e+9) | 1.02 (0.93 - 1.11) | 0.77 (0.49 - 1.22) |
| **MCI - CN** | 0.94 (0.01 - 74.86) | 1.02 (0 – 1.01e+9) | 0.74 (0.53 - 1.03) | 0.58 (0.17 - 1.97) |
| **MCI - Dementia** | 1.02 (0.97 - 1.07) | 0.96 (0.72 - 1.27) |  |  |
| **MCI - Death** | 0.08 (0 - 5.19) | 2.35 (0.19 - 28.63) | 1.06 (0.97 - 1.16) | 0.89 (0.51 - 1.55) |
| **Dementia - Death** | 0.99 (0.92 - 1.06) | **0.65 (0.46 - 0.9)*** |  |  |

*p < .05;. CN: Cognitively normal; MCI/Dementia: Mild cognitive impairment or Dementia; MCI-TB: Test-based mild cognitive impairment; ^†^per unitary increase in the ANU-ADRI; ^‡^per SD increase in EV-GRS; all estimates are from models adjusting for the ANU-ADRI and EV-GRS
